# Unraveling the Host Plant Alternation of *Cacopsylla pruni* – Adults but Not Nymphs Can Survive on Conifers Due to Phloem/Xylem Composition

**DOI:** 10.3389/fpls.2018.00484

**Published:** 2018-04-13

**Authors:** Jannicke Gallinger, Jürgen Gross

**Affiliations:** ^1^Laboratory of Applied Chemical Ecology, Federal Research Centre for Cultivated Plants, Institute for Plant Protection in Fruit Crops and Viticulture, Julius Kühn-Institut, Dossenheim, Germany; ^2^Department of Plant Chemical Ecology, Technical University of Darmstadt, Darmstadt, Germany

**Keywords:** phloem, chemical composition, psyllid, development, overwintering, host alternation, migration, conifer

## Abstract

Plant sap feeding insects like psyllids are known to be vectors of phloem dwelling bacteria (‘*Candidatus* Phytoplasma’ and ‘*Ca*. Liberibacter’), plant pathogens which cause severe diseases and economically important crop damage. Some univoltine psyllid species have a particular life cycle, within one generation they alternate two times between different host plant species. The plum psyllid *Cacopsylla pruni*, the vector of European Stone Fruit Yellows (ESFY), one of the most serious pests in European fruit production, migrates to stone fruit orchards (*Prunus* spp.) for mating and oviposition in early spring. The young adults of the new generation leave the *Prunus* trees in summer and emigrate to their overwintering hosts like spruce and other conifers. Very little is known about the factors responsible for the regulation of migration, reasons for host alternation, and the behavior of psyllids during their phase of life on conifers. Because insect feeding behavior and host acceptance is driven by different biotic factors, such as olfactory and gustatory cues as well as mechanical barriers, we carried out electrical penetration graph (EPG) recordings and survival bioassays with *C. pruni* on different conifer species as potential overwintering hosts and analyzed the chemical composition of the respective plant saps. We are the first to show that migrating psyllids do feed on overwintering hosts and that nymphs are able to ingest phloem and xylem sap of coniferous trees, but cannot develop on conifer diet. Analyses of plant saps reveal qualitative differences in the chemical composition between coniferous trees and *Prunus* as well as within conifer species. These differences are discussed with regard to nutritional needs of psyllid nymphs for proper development, overwintering needs of adults and restriction of ‘*Ca.* P. prunorum’ to *Prunus* phloem.

## Introduction

Phloem and xylem tissue enables plants to allocate their resources from sources to sinks and distribute phytohormones to regulate physiological processes. Especially the phloem is rich in nutrients (Douglas, 2006), making it a suitable food source for sap-sucking insects. Although mechanical barriers like sclerenchymatous fibrous rings are able to hinder phloem-feeders from reaching the vascular bundles (George et al., 2017), the phloem is poorly chemically defended (Douglas, 2006). Since decades studies focused on the chemical composition of phloem sap and the nutrition of phloem-feeding insects. Most work was done in the field of crops, such as rice (Fukumorita and Chino, 1982), broad bean, clover, and peas (Sandström and Pettersson, 1994; Wilkinson and Douglas, 2003) and their pests (especially aphids), because of the economic importance and the role of aphids as model organisms. Information about the composition of phloem and xylem sap of coniferous plants is rare. Ziegler and Mittler (1959) extracted phloem sap from *Picea abies* by stylectomy and found sucrose as the only sugar in paper chromatography analysis. Later studies focused on induced defense mechanisms in bark phloem after bark beetle attack (Rohde et al., 1996), food quality of needles (Schopf et al., 1982; Fisher and Fisher, 1987) and impact of air pollution on nutrition of conifers (Zedler et al., 1986; Kainulainen et al., 1993). These studies give an impression of which metabolites could be found in plant sap of coniferous trees, but compounds were extracted from whole plant tissue (bark resp. needles). More explicit knowledge about plant sap composition is important for a better understanding of the biology of phloem-feeding insects that migrate between two different host plant species, e.g., psyllids (Hemiptera: Psyllidae).

Psyllids or jumping plant lice are plant sap feeding insects encompassing more than 3000 species. Most of them are oligophagous and use perennial dicotyledonous angiosperms as host plants for reproduction (Hodkinson, 2009; Mayer et al., 2009, 2011). In the genus *Cacopsylla* two different strategies can be observed: There are polyvoltine species reproducing and feeding exclusively on the same host plant and univoltine species with an obligate alternation of two host plants (Ossiannilsson, 1992; Hodkinson, 2009). The latter migrate between their reproduction host plants (respective fruit crops) and their overwintering host plants (conifers) (Mayer and Gross, 2007; Mayer et al., 2011). For identifying their particular host plants for feeding and reproduction, volatile signals are used in many species during migration (Soroker et al., 2004; Gross and Mekonen, 2005; Mayer et al., 2008a,b, 2009; Weintraub and Gross, 2013).

The plum psyllid, *Cacopsylla pruni* is the only known vector of one of the most serious pests in European fruit production, the cell wall lacking bacterium ‘*Candidatus* Phytoplasma prunorum’ (Carraro et al., 1998). The phloem dwelling bacterium induces the European Stone Fruit Yellows (ESFY) (Seemüller and Schneider, 2004). Because infected trees yield poorly and die quickly, this plant disease causes high economic losses in European fruit production every year. So far no curative approach was found against this disease. Unfortunately, it is not possible to cultivate this obligate cell parasite outside of the host plant or vector, which hampers research toward a cure. Therefore, the only measure to inhibit infection of stone fruit orchards is to prevent invasion of the vector insect, as *C. pruni* alternates between *Prunus* spp. and coniferous trees during its life cycle. After reproduction and development on *Prunus* spp., the young adults (emigrant stage) emigrate and spend the rest of the year on spruce and other conifers (Thébaud et al., 2009; Jarausch and Jarausch, 2016). In early spring they return to *Prunus* spp. for reproduction (remigrant stage). Very little is known about the reason for migration and feeding behavior of psyllids during their life on conifers (Thébaud et al., 2009). To date it remains unclear whether overwintering psyllids actually feed on conifers. Former experiments with the closely related hawthorn psyllid *Cacopsylla melanoneura* failed, although the maintenance of body condition and level of hydration suggested feeding (Jackson et al., 1990). Because it was shown that adult *C. pruni* did not survive the winter on one of their reproduction hosts *Prunus spinosa* (Carraro et al., 2002; Thébaud et al., 2009), and that some migrating species including *C. pruni* already start migration to their overwintering host during summer (Mayer and Gross, 2007; Mayer et al., 2009; Jarausch and Jarausch, 2016), we hypothesize that *C. pruni* needs to feed on overwintering host plants during this long period and therefore needs to leave deciduous *Prunus* trees to migrate to evergreen conifers, which show yearlong photosynthesis and phloem activity. On the other hand, reproduction on coniferous trees could be impossible for *C. pruni*, forcing them to migrate back to *Prunus*. A better knowledge of the vector biology is needed to develop new control strategies against vector insects and bacterial pathogens (Gross and Gündermann, 2016; Perilla-Henao and Casteel, 2016).

Here, we studied the feeding behavior of adults and nymphs on several conifer species as well as *Prunus domestica*, and conducted bioassays to unveil *C. pruni*’s ability to survive and develop on plant sap of overwintering hosts. Furthermore, we extracted the phloem/xylem sap of both *Prunus* spp. and conifers and analyzed sugars and organic acids including amino acids.

## Materials and Methods

### Insects

*Cacopsylla pruni* remigrants (overwintered adults) were caught by beating tray method from *Prunus domestica* trees located at the experimental field of the Julius Kühn-Institut in Dossenheim, Germany and at an experimental orchard of Dienstleistungszentrum Ländlicher Raum Rheinpfalz, Neustadt an der Weinstrasse, Germany in spring 2017. Psyllids were maintained on *Prunus* trees (cv. GF655/2 and *Prunus spinosa*) in cages housed in a climate chamber at 20°C during photophase and 16°C during scotophase (L16:D8). After mating and oviposition the field captured adults were transferred to cages with fresh plants. For survival experiments about 200 fifth instar nymphs were gently transferred to a new *P. domestica* (cv. Wavit) tree and emerged adults (emigrants) were collected daily.

### Plants

Four conifer species, *Abies alba* (Silver fir), *Larix decidua* (European larch), *Picea abies* (Norway spruce), and *Pinus sylvestris* (Scots pine), and the *P. domestica* cultivar Wavit were used for experiments. Plants were grown under natural conditions in an insect safe environment. Hexythiazox (Ordoval, BASF, Ludwigshafen am Rhein, Germany) and Fenpyroximate (Kiron, Cheminova Deutschland GmbH & Co. KG, Stade, Germany) were applied once to *P. domestica* plants in April 2017 to prevent infestation with spider mites.

### EPG-Recordings

To investigate whether *C. pruni* adults and nymphs feed on coniferous trees in general, the electrical penetration graph technique (EPG) was applied. EPGs were recorded using an 8 channel amplifier (model Giga-8d, EPG-Systems, Wageningen, Netherlands). Data acquisition and analysis was performed with Stylet+ software (EPG-Systems). To connect the psyllids to a copper electrode, a piece of fine gold wire (18 μm) was attached to the pronotum with a small droplet of water based silver glue (EPG-Systems). The electrode was attached to an EPG probe and the reference electrodes were placed in the soil of the test plants. Feeding behavior of *C. pruni* male and female emigrants (minimum age 6 weeks) was recorded in a climate chamber at 10°C with 60–65% RH for 16 h and of fifth instar nymphs (about 6 weeks old) at 20°C under the same conditions. Plants and insects were housed in a grounded self-constructed Faraday cage during recordings. Recordings were replicated 10 times for nymphs on each *P. abies*, *A. alba*, and *P. domestica* (cv. Wavit). Feeding behavior of emigrants was recorded on *P. sylvestris* (4 males and 6 females), *P. abies* (6 males and 4 females), *A. alba* (5 males and 5 females), and *L. decudia* (6 males and 4 females). To ensure that emigrants used for EPG recordings were not repelled by conifers (due to their developmental stage), *C. pruni* adults were caged with *P. abies* and *A. alba* twigs one day prior recordings and only emigrants which were found on conifer twigs were chosen for the experiment. Recordings were examined for occurrence of stylet penetration and waveforms indicating phloem and xylem uptake according to Bonani et al. (2010) and Civolani et al. (2011).

### Bioassays

#### Survival

Survival of emigrants was studied on *P. abies*, *A. alba*, and *P. domestica* cv. Wavit plants. Transparent plastic cups (0.5 l capacity) were used as cages. The bottom of each cup was replaced by gauze for venting. A hole was punched into the lids to attach the cups on twigs of living plants. The lid was sealed with self-made modeling clay (composed of 42.6% water, 42.6% flour, 3.2% sunflower oil, 10.6% salt, and 1.1% citric acid) and five newly emerged emigrants (<24 h) were released in each cup. Living individuals were recorded daily over a period of 40 days. Additionally, the mortality of emigrants in the same type of cups, but without food supply (control), was observed. The experiment was replicated eight times for every plant species and five times without plants (control) under rearing conditions.

#### Development

For developmental experiments *C. pruni* nymphs of second and third instar were gently transferred with a fine brush from rearing plants to twigs with young flush of *P. abies*, *A. alba*, or *P. domestica* cv. Wavit, respectively. On each plant, five nymphs were caged in insect rearing sleeves (40 cm × 20 cm, 100 × 80 mesh/square inch, MegaView, Taiwan). The experiment was replicated seven times on each conifer species and five times on cv. Wavit. Experimental plants were housed under rearing conditions in a climate chamber. After 21 days cages were controlled consistently once a week for hatched *C. pruni* adults (emigrants). After 56 days all cages were opened and checked for living nymphs.

### Xylem and Phloem Sap Sampling

Phloem and xylem saps were collected in June 2017 using modified centrifugation technique according to Hijaz and Killiny (2014). The twigs from young flush from *P. domestica* (cv. Wavit) and conifer species *P. abies, A. alba, L. deciduas*, and *P. sylvestris* were sliced into 2–3 cm pieces with a clean scalpel. The bottom of a 0.5 ml Eppendorf tube was removed with a razor blade and twig pieces were placed into the tube. The tube was immersed in a 1.5 ml tube. For collecting the phloem and xylem sap, the tubes were centrifuged at 12.000 rpm at 4°C for 10 min. The collected samples were stored at -80°C up to analysis. In the following, this collected mixture of phloem and xylem sap is referred as plant sap.

### Plant Sap Derivatization

The sap samples were derivatized with methyl chloroformate (MCF) to focus the GC-MS analysis on amino and other organic acids (Smart et al., 2010). An aliquot of 20 μl plant sap was mixed with 180 μl sodium hydroxide (1 M) in a silanized glass vial. Then 167 μl methanol and 34 μl pyridine were added, followed by 20 μl MCF. The sample was vortexed for exactly 30 s, additionally 20 μl MCF were added and the sample was mixed again for 30 s. To extract the alkylated derivatives 150 μl chloroform were added to each sample and mixed for another 10 s. A 200 μl aliquot of sodium bicarbonate solution (50 mM) was added and mixed for 10 s again. After a double meniscus was formed, the aqueous phase was discarded and a few milligrams of anhydrous sodium sulfate were added to the organic layer to bind the remaining water. The supernatant was transferred to a GC-MS vial with a glass insert.

For the derivatization with trimethylsilyl (TMS) 5 μl aliquots of the sap samples were added to 60 μl of an internal standard solution (Ribitol in ultrapure water) and dried under nitrogen stream (Reacti-Vap, Thermo Fisher Scientific Inc., Waltham, MA, United States). Samples were derivatized by adding 70 μl methoxyamine hydrochloride solution (MOX) in pyridine (2%) and allow to incubate for 90 min at 37°C stirring at adjustment of 7 (Reacti-Therm, Thermo Fisher Scientific Inc.). 90 μl of N-methyl-N-(trimethylsilyl)trifluoroacetamide (MSTFA) were added and the silylation was allowed to react for 60 min at 37°C stirring at adjustment of 7 (Reacti-Therm, Thermo Fisher Scientific Inc.). The supernatant was transferred to a GC-MS vial with a glass insert.

### Chemical Analysis

Derivatized samples were analyzed by gas chromatography coupled with mass spectrometry (GC-MS) using a PerkinElmer Clarus R 680 GC system coupled to a PerkinElmer quadrupole inert mass selective detector for molecular structure analysis. A non-polar Elite-5MS (Crossbond 5% diphenyl-95% dimethyl polysiloxane, PerkinElmer) capillary column (30 × 0.25 mm id × 0.25 μm film thickness) was used for GC separation. Carrier gas flow rate (Helium, Linde, Germany) was about 5 ml/min (column head pressure 150 kPa). Injection of 1 μl of the samples derivatized with MCF was done at 290°C injector temperature with a split flow of 1 ml/min. The initial oven temperature of 70°C was held for 3 min, followed by a temperature increase of 20°C/min up to 240°C held for 3.5 min and a further increase to 300°C at a rate of 20°C/min. The final temperature of 300°C was held for 2 min. The GC temperature program to analyze samples after silylation was as follows: the initial oven temperature of 80°C was held for 3 min, followed by an increase of 5°C/min up to 320°C. The final temperature of 320°C was held for 4 min. One microliter of each sample was injected at 220°C with a split flow of 5 ml/min. Transfer line and ion source temperatures were set to 250°C and 180°C, respectively. The quadrupole mass detector was operated in electron-impact (EI) mode at 70 eV. All data were obtained by collecting the full-scan mass spectra within the range of 35–550 m/z. Blank samples, reference standards and mixtures of alkanes (C8–C20 and C10–C40) were analyzed additionally according to both methods.

### Identification and Quantification With AMDIS

GC-MS chromatograms were analyzed using “Automated Mass spectral Deconvolution and Identification System” (AMDIS, V. 2.71; National Institute of Standards and Technology NIST, Boulder, CO, United States). Detected compounds were identified by comparing characteristic ion fragmentation patterns, retention times and retention indices with standard compounds according to Weintraub and Gross (2013). For quantification, the peak areas were integrated after deconvolution with AMDIS. Identification criteria were applied as follows: match factor had to be ≥80% and the relative retention index deviation ≤5% from reference value. The settings for deconvolution were: component width: 32; adjacent peak subtraction: one; resolution: medium; sensitivity: medium; shape requirements: high; level: strong; maximum penalty: 20, and ‘no RI in library’: 20. Methionine, threonine, and serin were only found in traces (match < 80) and were therefore excluded from the analysis. Relative proportions of amino and organic acids were calculated by setting the sum of the selected compounds as 100%. Proportions of detected compounds after TMS derivatization were normalized to internal standard.

### Chemicals and Standards

Alanine, aspartic acid, cysteine, glutamic acid, histidine, leucine, lysine, proline, threonine, tryptophan, valine, salicylic acid, pyridine, methanol, chloroform, methyl chloroformate (MCF), sodium bicarbonate, sodium sulfate, methoxyamine, ribitol, myo-inositol, xylose, pinitol, and iso-leucine were purchased from Sigma-Aldrich Chemie GmbH (Munich, Germany). Arginine and phenylalanine were purchased from SERVA Electrophoresis GmbH (Heidelberg, Germany). Glycine, methionine, serine, malic acid, caffeic acid, succinic acid, arabinose, and saccharose from Carl Roth GmbH & Co. KG (Karlsruhe, Germany). Asparagine, mannitol, glucose, and galactose from Merck KGaA (Darmstadt, Germany). Sorbitol and glutamine from AppliChem GmbH (Darmstadt, Germany). MSTFA from Macherey-Nagel GmbH & Co. KG (Düren, Germany). Citric acid was purchased from Acros Organics (Thermo Fisher Scientific, Geel, Belgium).

### Statistical Analysis

Statistical analysis was done in R version 3.4.2 “Short Summer” (R Core Team, 2017). Visualizations were conducted with the ggplot2 package (Wickham, 2009). Death hazard from *C. pruni* emigrants on different host plants were compared by Cox’s proportional hazard regression through likelihood ratio test. Efron approximation was used for tie handling. The proportional hazards assumption for Cox regression model fit was confirmed using the *cox.zph* function of the survival package (Therneau, 2017). Non-metric multidimensional scaling (NMDS) plots were used to visualize Bray–Curtis dissimilarities of the chemical composition of xylem and phloem between plant species. NMDS was performed using the *metaMDS* function from vegan package (Oksanen et al., 2017). Scaling was standardized by Wisconsin double standardization. Significantly (*p* < 0.01, *N* = 10000) influential factors (chemical compounds) were plotted as arrows in NMDS plots. Dissimilarity matrix was calculated to test for discrimination of plant species by Permutational Multivariate Analysis of Variance (PERMANOVA). Additionally, the dispersion of groups was tested for multivariate homogeneity (PERMDISP).

## Results

### EPG-Recordings

**FIGURE 1:**
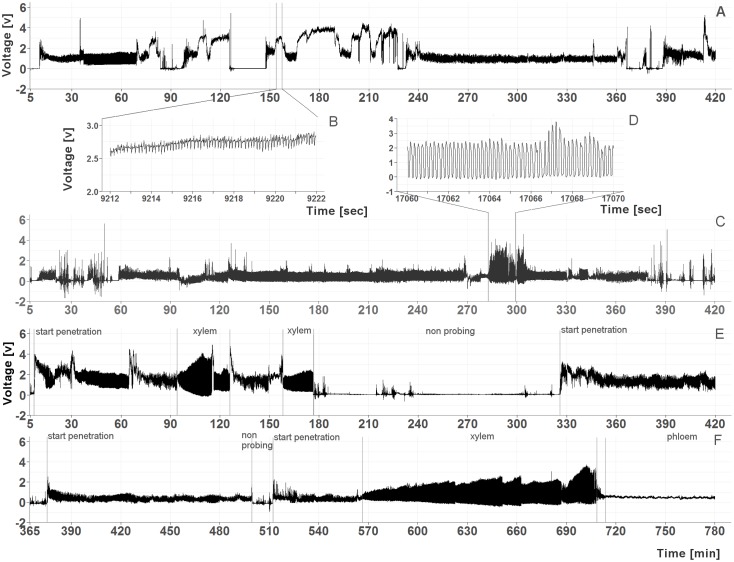
**(A–D)** Examples of EPG recordings from *C. pruni* nymphs (5th instar) on spruce **(A)** with a detailed magnification of phloem phase waveform **(B)** and on fir **(C)** with a detailed magnification of the waveform of xylem feeding **(D)**. **(E,F)** Examples of recordings from a female *C. pruni* emigrant on larch **(E)** and a male emigrant on fir **(F)** with marked penetration and feeding phases.

To determine if *C. pruni* feeds on overwintering hosts (conifers), feeding behavior of emigrants was recorded on potential host plants. The recordings revealed that both male and female emigrants fed on plant saps of all four offered conifers: *P. abies*, *A. alba*, *P. sylvestris*, and *L. decudia*. Recordings from nymphs of *C. pruni* showed that they were also able to feed on *P. abies* and *A. alba* (**Figure 1**).

### Bioassays

#### Survival

**FIGURE 2:**
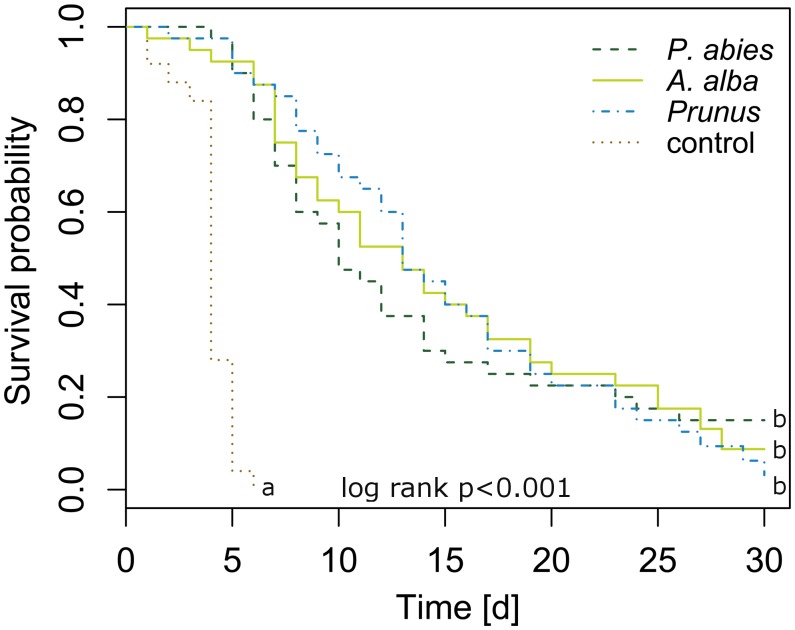
Kaplan–Meier curves visualizing the survival of newly emerged emigrants caged on *P. abies* (*n* = 40), *A. alba* (*n* = 40), *P. domestica* cv. Wavit (*n* = 40), or in cages without a plant (control, *n* = 25). Letters indicate significant differences between survival curves (likelihood ratio = 81.76, *df* = 3, *R*^2^ = 0.431, *p* < 0.001).

Newly emerged *C. pruni* emigrants survived on *P. abies* and *A. alba* as long as on *P. domestica* cv. Wavit (**Figure 2**). The Cox regression model showed that death hazard differed significantly between host plants and controls without food supply (likelihood ratio = 81.76, *df* = 3, *R*^2^ = 0.431, *p* < 0.001). Death hazard for emigrants fed on *P. domestica* cv. Wavit did not differ from *P. abies* (likelihood ratio = 81.76, *df* = 3, *R*^2^ = 0.431, *p* = 0.803) and *A. alba* (likelihood ratio = 81.76, *df* = 3, *R*^2^ = 0.431, *p* = 0.846). Emigrants on all three potential host plant species had a significant lower death hazard than psyllids without food (control). The hazard ratio was reduced by 97, 97, and 96% if *C. pruni* was allowed to feed on *P. abies*, *A. alba*, or *P. domestica* cv. Wavit, respectively.

#### Development

**FIGURE 3:**
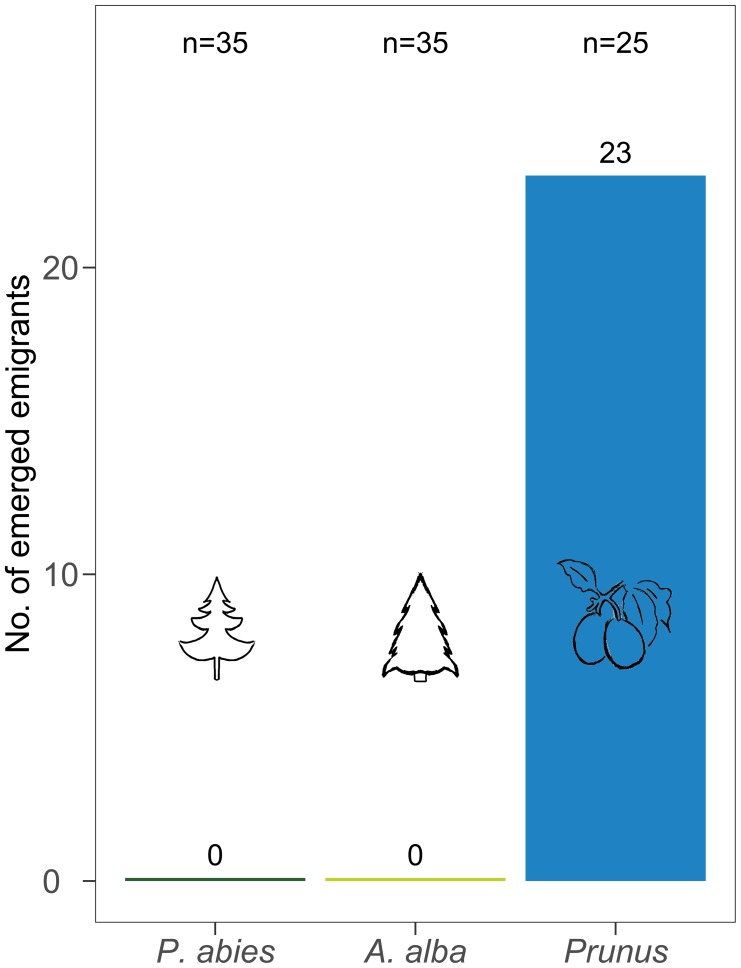
Number of emerged *C. pruni* emigrants from nymphs (2nd instar) on *P. abies*, *A. alba*, and *P. domestica* cv. Wavit.

After 56 days 92% of the *C. pruni* nymphs on *P. domestica* cv. Wavit emerged while none of the nymphs developed neither on *P. abies* nor *A. alba* (**Figure 3**). As no living nymphs could be found on the coniferous trees, we conclude that they all died in nymphal stage.

### Chemical Composition of Phloem and Xylem Content

**FIGURE 4:**
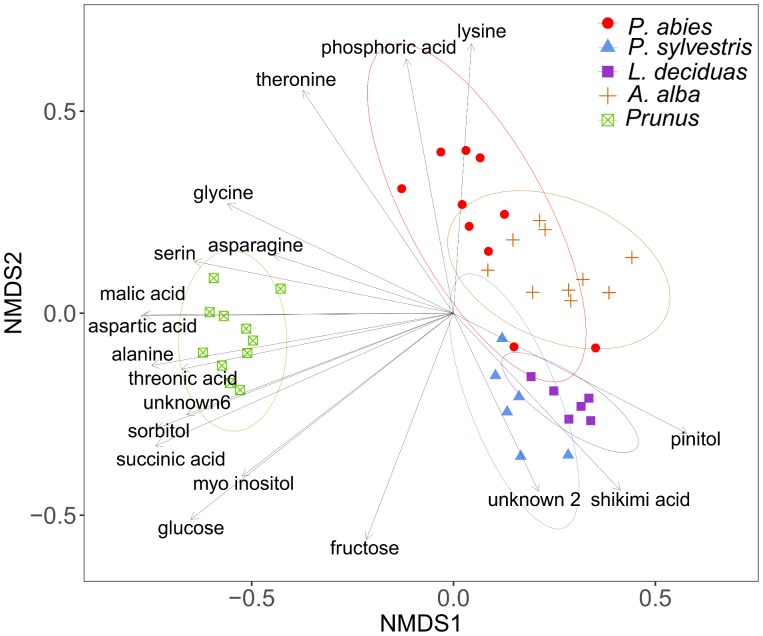
Visualization of Bray–Curtis dissimilarities with non-metric multidimensional scaling (NMDS) plots (stress = 0.14) of plant sap samples from spruce (*n* = 10), pine (*n* = 6), larch (*n* = 6), fir (*n* = 10), and *P. domestica* cv. Wavit (*n* = 11) after methoximation followed by trimethylsilylation.

Plant species differed significantly in the chemical composition of sugars and other compounds detected by GC-MS analysis after TMS derivatization of plant sap (PERMANOVA, *df* = 4, *R*^2^ = 60.83, *N* = 10000, *P* < 0.001). The dispersions differed not significantly between the groups (PERMDISP, *df* = 4, *F* = 0.42, *N* = 10000, *P* > 0.05), confirming that separation of species was due to their location. The NMDS plot illustrates the differences of chemical profiles (**Figure 4**).

**FIGURE 5:**
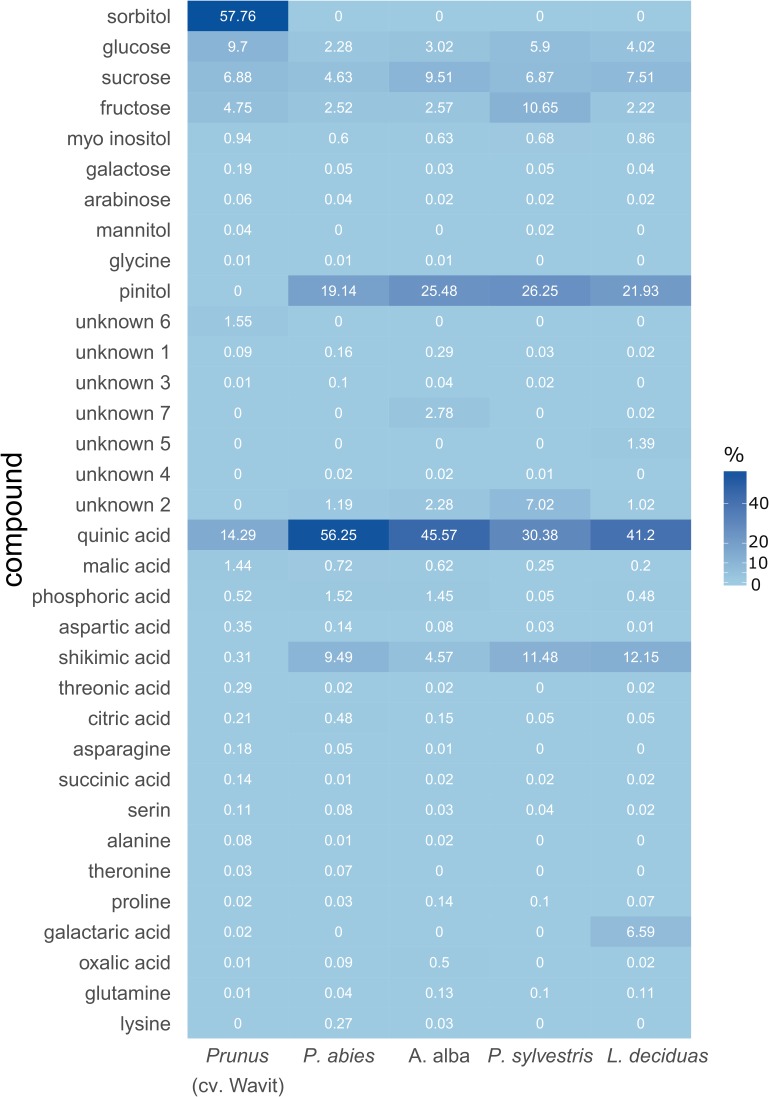
Composition of sugars and acids in vascular bundle content of *P. domestica* cv. Wavit (*n* = 11), spruce (*n* = 10), fir (*n* = 10), pine (*n* = 6), and larch (*n* = 6). Plant sap was collected by centrifugation and derivatized by trimethylsilylation after methoximation. Dark blue indicates a high relative abundance of the components, light blue a low abundance. Numbers are mean values of relative abundance.

Plant saps from *P. domestica* trees contained a high amount of sorbitol. This sugar alcohol constituted about 58% of the plant sap from *P. domestica* cv. Wavit but was not detected in samples from coniferous trees (**Figure 5**). In contrast, pinitol was exclusively found in plant sap from conifers. However, the most abundant component was quinic acid in all conifer samples (**Figure 5**). The relative abundance of quinic acid ranged from 30% in pine to 56% in spruce. Sap samples of *P. domestica* were composed of 80% sugars and sugar alcohols and 18% acids, whereas spruce, fir, pine, and larch samples consisted of 29, 41, 50, and 36% sugars and sugar alcohols and 69, 53, 43, and 61% acids, respectively.

**FIGURE 6:**
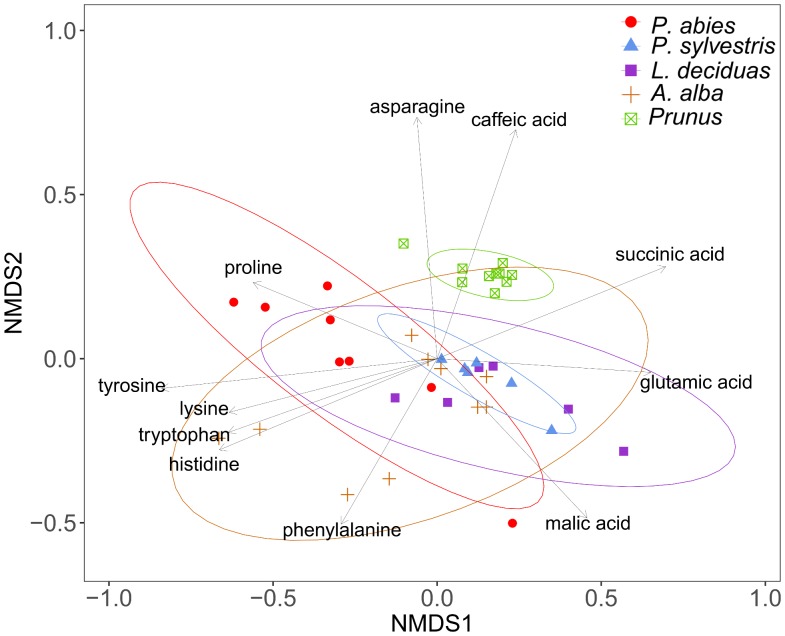
Visualization of Bray–Curtis dissimilarities with NMDS plots (Stress: 0.13) of plant sap samples from spruce (*n* = 8), pine (*n* = 6), larch (*n* = 6), fir (*n* = 10), and *P. domestica* cv. Wavit (*n* = 10) derivatization with methyl chloroformate.

**FIGURE 7:**
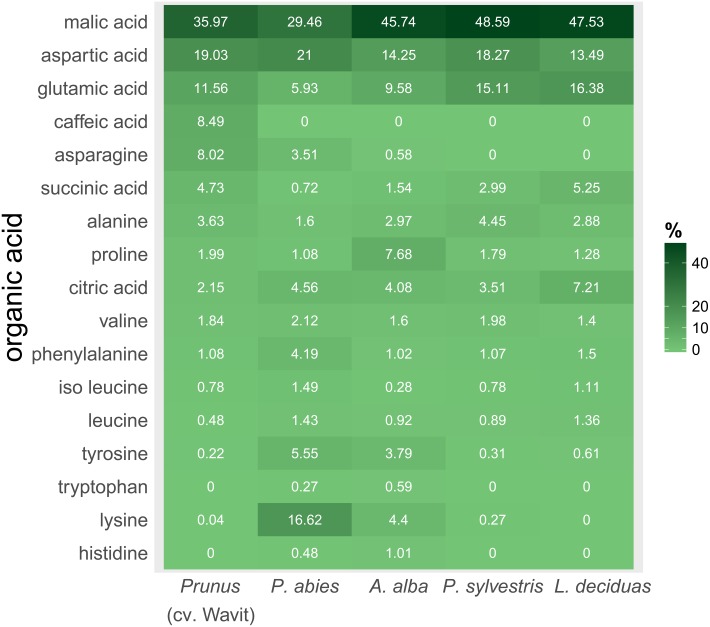
Composition of organic acids in vascular bundle content of *P. domestica* cv. Wavit (*n* = 10), spruce (*n* = 8), fir (*n* = 10), pine (*n* = 6), and larch (*n* = 6). Plant sap was collected by centrifugation and derivatization with methyl chloroformate. Dark green indicates a high relative abundance of a respective organic acid, light green a low abundance. Numbers are mean values of relative abundance.

The composition of amino acids and other organic acids differed significantly between the plant species (PERMANOVA, *df* = 4, *R*^2^ = 46.85, *N* = 10000, *P* < 0.001). The dispersions between the groups also differed significantly (PERMDISP, *df* = 4, *F* = 3.96, *N* = 10000, *P* < 0.01), indicating that the separation of the plant species could be effected by different variation within species (**Figure 6**). The NMDS plot shows caffeic acid and asparagine contributing to the separation of *P. domestica* cv. Wavit from coniferous trees (**Figure 6**). Caffeic acid was exclusively found in *P. domestica* cv. Wavit, while asparagine was more abundant in *P. domestica* cv. Wavit as in *P. abies* and *A. alba* (**Figure 7**).

The main organic acid component in the plant sap of all tested plant species was malic acid (29–48%). Aspartic acid was the second most abundant component in all plants, except in larch which contained more glutamic acid. Differences between the plant species were detected concerning the relative amounts of lysine in the plant sap composition. Lysine represented about 17% of the sap samples of spruce trees and was the third most abundant component in those trees, as glutamic acid was in fir (10%), pine (15%), and *P. domestica* cv. Wavit (12%) (**Figure 7**). Cysteine, methionine, and threonine were under detection limits in all samples. The NMDS plots indicate the responsibility of the essential amino acids tyrosine, tryptophan, lysine, and histidine on the separation of spruce and fir from *P. domestica* cv. Wavit (**Figure 6**).

## Discussion

Electrical penetration graph recordings showed that *C. pruni* emigrants and nymphs are able to feed on the plant saps of spruce, pine, larch, and fir. EPGs recorded from 5th instar nymphs prove that nymphs are not repelled by metabolites of coniferous plants and able to reach the phloem and xylem tissue with their stylet. The question arises why *C. pruni* migrates to *Prunus* for reproduction when their progeny is able to ingest food from conifers. We suggest that there is no change in host acceptance of nymphs between different instars, but nutritional needs could change between nymphal development stages. Therefore we investigated the emergence of adults starting from the earliest possible instar (2nd). Because the impact of low food quality or inhibitory components may accumulate and negative influence raise over time, 5th instar nymphs may able to compensate a short period on a non-optimal diet while early instars would suffer more from low food quality than later ones. But it is of crucial importance, whether *C. pruni* is able to fully develop from egg to adult stage on coniferous trees. Bioassays revealed that adult psyllids survived on coniferous trees, while nymphs did not develop and died, although they were able to ingest plant sap from conifer needles. Thus, the chemical composition of the respective conifer saps influences the nymphal survival and development. Therefore the plant saps of overwintering hosts were subsequently analyzed and compared to sap content of their reproduction host plant (*P. domestica*).

The GC-MS analysis revealed enormous differences in the chemical compositions of plant sap of the Rosacea species *P. domestica* cv. Wavit and the four studied conifer species. Especially the lack of sorbitol in all four conifers as well as the high amount of quinic acid and pinitol (which was not detected in *Prunus* trees) could be challenging for phloem feeding insects, which alternate between Rosacea and conifers during their life cycle. Even though it was known that spruce needles contain quinic acid, shikimic acid, fructose, glucose, sucrose, and pinitol (Schopf et al., 1982), to date it was unclear, in which proportions they occur in the phloem and xylem sap of coniferous trees, and how their proportions differ between tree species.

Until today it was a widespread belief that conifers are used by migrating *Cacopsylla* species like *C. pruni*, *C. picta*, and *C. melanoneura* for shelter during winter time, exclusively (Burckhardt et al., 2014; Jarausch and Jarausch, 2016). In the presented study we were able to show for the first time, that conifers are not only shelter plants for migrating species belonging to the genus *Cacopsylla*, but also an important food resource enabling their overwintering. Thus, the term “shelter plant” should hereafter be replaced by “overwintering host” or just “alternate host” plant.

Due to the lack of knowledge that psyllids feed on conifers, the effect of coniferous phloem constituents like quinic acid, shikimic acid, and pinitol on psyllid feeding behavior and development was not studied before. Pinitol is a cyclic polyol, which serves as osmoprotectant and is involved in a broad spectrum of physiological processes in plants (Chiera et al., 2006; Kordan et al., 2011; Saxena et al., 2013). It is found in conifers, legumes (Fabaceae) and Caryophyllales such as *Simmondsia chinensis* (Angyal and Macdonald, 1952; Dittrich and Korak, 1984; Guo and Oosterhuis, 1995; Chiera et al., 2006). D-pinitol induces oviposition of the Grass Yellow Butterfly *Eurema mandarina* (Mukae et al., 2016). However, an influence of pinitol from the phloem of alfalfa on phloem-feeding pea aphid could not be found (Campbell and Binder, 1984).

There is evidence, that psyllid adults and nymphs are tolerant to high osmotic pressures of their diets (Hall et al., 2010; Russell and Pelz-Stelinski, 2015). Therefore, we hypothesize no negative influence of pinitol on *C. pruni*, even if it occurs in high amounts in overwintering hosts. Quite the contrary, pinitol could act as mechanism of protection against freezing stress, as shown for other polyols (Bale, 2002). The freezing temperature of the green spruce aphid is reduced in the presence of mannitol in aphid hemolymph (Parry, 1979). Whiteflies accumulate sorbitol for thermo- and osmoprotection (Hendrix and Salvucci, 1998). Sømme (1965) found an accumulation of sorbitol in overwintering eggs of European red mite (*Panonychus ulmi*).

We found that sorbitol is the most abundant component in sap samples of *P. domestica* cv. Wavit, which is in accordance with the fact that sorbitol is most often found in Rosacea (Loescher, 1987). Sorbitol is also known to be accumulated in the phloem of apple trees (Bieleski, 1969) and is the most abundant soluble sugar in the phloem of pear and apple fruits (Zhang et al., 2004, 2014). Nevertheless, adult *C. pruni* can tolerate high amounts of sorbitol or pinitol in their diet. EPG recordings suggest that *C. pruni* (both adults and nymphs) also ingest xylem content (unpublished results), which could be a regulatory reaction to reduce the phloem’s high osmotic pressure by dilution. Pompon et al. (2011) showed that aphids ingest more xylem sap after feeding on high concentrated sucrose diets to compensate osmotic unbalance. Moreover, for nymphal development the availability of amino acids (especially essential amino acids) could be of higher importance, as nitrogen content of food is an important limiting growth factor for phytophagous insects (Douglas, 2006). In accordance with Douglas (1993) we found asparagine besides aspartic acid and glutamic acid as one of the most abundant amino acids in young leaves of *Prunus*, while we found only low concentrations of glutamine in *Prunus* flush leaves. All plant species contained only low concentrations of the essential amino acids histidine, isoleucine, leucine, lysine, methionine, phenylalanine, threonine, tryptophan, and valine. To compensate for low quality of nitrogen in plant saps phloem feeders harbor microsymbionts (Douglas, 2006). Many psyllid species harbor the bacterial endosymbiont *Carsonella ruddii*, which provides its host with essential amino acids (Thao et al., 2000). Also representatives of the genus *Wolbachia*, *Arsenophonus* and other *Enterobacteriaceae* were found in psyllids (Baumann, 2005). Although the microsymbionts harbored by *C. pruni* are unidentified, differences in the symbiont community in adults and nymphs were not expected, because vertical transmission of endosymbionts was shown for many species. Furthermore, recent studies indicated the transovarial transmission of *Arsenophonus* in *Cacopsylla pyricola* (Cooper et al., 2017).

We suggest that the inability of *C. pruni* nymphs to develop on coniferous trees is due to differences in organic acid availability. The caffeic acid, which is exclusively found in cv. Wavit, could play a key role in host acceptance of *C. pruni* and maybe act as a phagostimulant. Caffeic acid was found in several stone fruits like peaches and plums, which are typical host plants of *C. pruni* (Carbonaro et al., 2002; Lombardi-Boccia et al., 2004). However, not all of the components responsible for the separation of cv. Wavit from the coniferous species need to be of biological relevance. To unravel which components are actually important for proper development or which ones may inhibit nymphal growth, feeding experiments with nymphs on artificial diets are crucial. The analysis of excreted honeydew could suggest important information on how psyllids process plant nutrients. This study also revealed differences between the plant saps of the investigated coniferous trees. Therefore, a detailed analysis of EPG recordings from nymphs on the different tree species could be needful to identify feeding stimulants or deterrents and will be investigated in future. This knowledge could be used for development of an artificial diet system for rearing of *C. pruni* and screening for potential toxins against psyllids (Jancovich et al., 1997; Hall et al., 2010). Interestingly, although some of the migrating psyllids like *C. pruni* harbor phloem-limited plant pathogenic bacteria (‘*Ca.* Phytoplasma’ or ‘*Ca.* Liberibacter’) and feed on conifers, the phytopathogens seem to be restricted to vector insects and their reproduction host plants (Gross, 2016). Because the genomes of Phytoplasma spp. lack metabolic genes but contain a lot of transporter systems, it is suggested that they depend strongly on the nutrition of their hosts (Oshima et al., 2004; Kube et al., 2008). Insight on the chemical composition of the phloem sap of host plants could support developing a culture media for phytoplasmas and may advance the research on phytoplasma diseases (Trivedi et al., 2016).

## Conclusion

No mechanical nor chemical border prevents *C. pruni* adults and nymphs from feeding on conifers. Emigrants feed and survive on their overwintering hosts. Nymphs can feed on, but are not able to develop on spruce and fir. This is likely due to strong differences in the compositions of organic acids and sugars between plant saps of conifers and *P. domestica*. Furthermore, feeding experiments with nymphs on artificial diets should reveal which components are responsible for successful development of *C. pruni*. Additionally, more insight on phloem sap composition could open up new possibilities for phytoplasma cultivation and pathogen research.

## Author Contributions

JGa and JGr designed the study, contributed to the interpretation of the data, approved the final version of the manuscript, and ensured the accuracy and integrity of the work. JGa conducted the experiments and analysis and wrote the first draft of the manuscript, which was revisited and edited by JGr. JGr supervised the project.

## Conflict of Interest Statement

The authors declare that the research was conducted in the absence of any commercial or financial relationships that could be construed as a potential conflict of interest.
